# Effects of Exposure, Diet, and Thermoregulation on Fecal Glucocorticoid Measures in Wild Bears

**DOI:** 10.1371/journal.pone.0055967

**Published:** 2013-02-14

**Authors:** Jeff Stetz, Kathleen Hunt, Katherine C. Kendall, Samuel K. Wasser

**Affiliations:** 1 Sinopah Wildlife Research Associates, Missoula, Montana, United States of America; 2 Department of Biology, University of Washington, Seattle, Washington, United States of America; 3 Northern Rocky Mountain Science Center, US Geological Survey, West Glacier, Montana, United States of America; University of Sydney, Australia

## Abstract

We examined fecal glucocorticoid (fGC) measures of nutrition and thermoregulatory demands on wild bears in Glacier National Park, Montana, and assessed how these measures changed in samples left in the field. Both ambient temperature and exposure can impact thermoregulation and sample degradation. Bear diets vary markedly with season, affecting body condition and thus fGC. We collected fecal samples during September and October, 2001, when ambient temperatures ranged from 30°C to −5°C. We collected half of each sample immediately and left the other half in its original location for 1–28 days. We used generalized linear models (GLM) to first predict fGC concentrations in fresh samples based on proxies of nutrition, ambient temperature, thermal exposure, and precipitation. These same covariates were then used to predict degradation-based differences in fGC concentrations between the paired sample halves. Variation in fGC was predicted by diet, Julian date, aspect, and the interaction between Julian date and aspect in both fresh and exposed samples. Cumulative precipitation was also a significant predictor of fGC concentrations in the exposed samples, independent of time, indicating that precipitation contributes to sample degradation but not enough to mask effects of other environmental factors on fGC concentrations. Differences between sample halves were only predicted by cumulative precipitation and exposure time; cumulative precipitation decreased, whereas exposure time increased, fGC concentrations in the exposed sample halves. Results indicate that fGC can provide reliable indices of nutrition and thermoregulatory demands in bears and that sample degradation impacts on these relations are minimal and can be virtually eliminated by controlling for cumulative precipitation over the estimated exposure times.

## Introduction

Fecal hormone analysis has become a widely used technique for measuring an animal’s endocrine status and can provide valuable information to conservation and monitoring programs. Fecal samples are often easily found and identified to the species level and can be collected without disturbing wildlife [1]. Analysis of hormones in these samples can provide a variety of stress, reproductive, and metabolic status measures that can be correlated with environmental pressures over time [2]–[10]. Noninvasive sample collection, however, often includes samples that have been exposed to variable environmental conditions for varying and unknown time periods. Understanding how time and exposure in the natural environment affect hormone degradation is a prerequisite to reliable interpretation of fecal hormone levels, particularly if the same natural conditions causing variation in hormone levels (e.g., ambient temperature) also promote hormone degradation [11], [12].

Physiological measures of climate-related thermoregulatory demands provide a case in point. Monitoring effects of climate change can be difficult because cumulative effects take place on large geographic scales over long time frames. Noninvasive physiological measures of thermoregulatory demands and associated impacts from habitat shifts in wildlife over large landscapes could provide a sensitive tool for early detection and monitoring of such impacts. Fecal glucocorticoids (fGC: cortisol, corticosterone, and their metabolites) could provide one such measure, having been shown to reflect physiological responses to ambient temperature and thermal exposure in mammals [13], [14]. Monitoring these impacts in nature can be difficult, however, because warmer ambient temperatures and environmental exposure may also hasten fGC degradation, and both temperature and exposure effects are likely to vary with time of year [15]. Nutritional status, which is likely to vary with time of year, can also be detected through fGC levels [2], [5], [10], [16], [17]. Yet, diet composition itself can impact steroid excretion rates [18]–[21]. Here, we ask: can we detect a biological relationship between thermoregulatory demands or nutrition and fGC concentrations in bears? How does fGC degradation change with time, temperature, and exposure? Are these degradation effects large enough to mask fGC biological indices of thermoregulation and nutrition?

We separate thermoregulatory from degradation-related effects on fGC levels in a study of grizzly bears (*Ursus arctos horribilis*) and American black bears (*U. americanus*) in Glacier National Park, MT. Julian date (estimating ambient temperature) plus aspect, slope, and elevation (collectively estimating thermal exposure), and precipitation at sample collection locations were used to predict thermoregulatory impacts by measuring fGC levels in fresh fecal samples at time 0 (i.e., samples <24 hours old) compared to fGC in those same samples left under the same natural field conditions for an additional, randomly assigned period of 1–28 days. Scat contents were also used in those samples to examine how fGC varies with diet/nutrition, as well as to separate diet impacts from effects of thermoregulation and degradation on fGC.

## Methods

### Study Area

**Figure 1:**
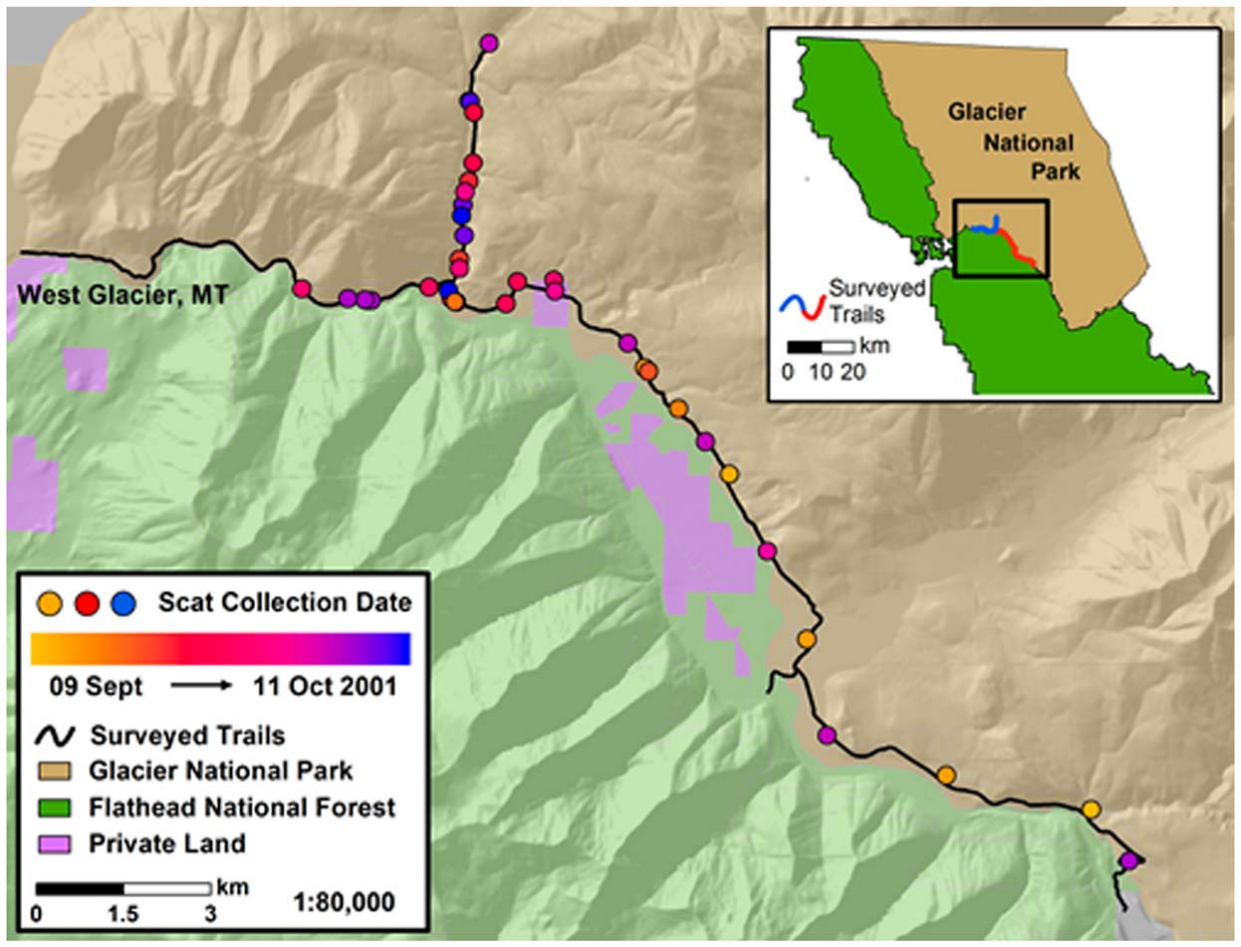
Map showing the location of surveyed trails and scats collected in Glacier National Park, Montana. Scats were collected during 09 Sept-11 Oct, 2001.

We collected bear scats from 2 sections of trail located near the southern boundary of Glacier National Park (GNP), MT (Fig. 1). Generally, these trails paralleled the park boundary with a mix of National Forest and private lands along the opposite side of the Middle Fork of the Flathead River. This area has very little human development; however, U.S. Highway 2 and the Burlington Northern Santa Fe railroad experience intermittent heavy human use. Our study occurred at the end of the tourist season when highway traffic volumes were well below the peaks experienced in mid-summer.

The study area was located just west of the Continental Divide, in a relatively moist climate reflecting a maritime influence. Elevation ranged from approximately 950 m to 1250 m. Surveyed trails ran mostly on westerly or southerly facing slopes through mature forest consisting largely of Douglas fir (*Pseudotsuga menziesii*), Engelmann spruce (*Picea engelmannii*), lodgepole pine (*Pinus contorta*), and western larch (*Larix occidentalis*).

Although GNP provides a high level of security for wildlife within its borders, bears are known to readily move outside the park and across the U.S. Highway 2/railroad corridor. Outside GNP bears are exposed to a variety of stressors related to human activities such as train and highway traffic, private residences and small public lodgings, and various forms of outdoor recreational and industrial activities such as logging.

### Sample Collection and Processing

**Figure 2:**
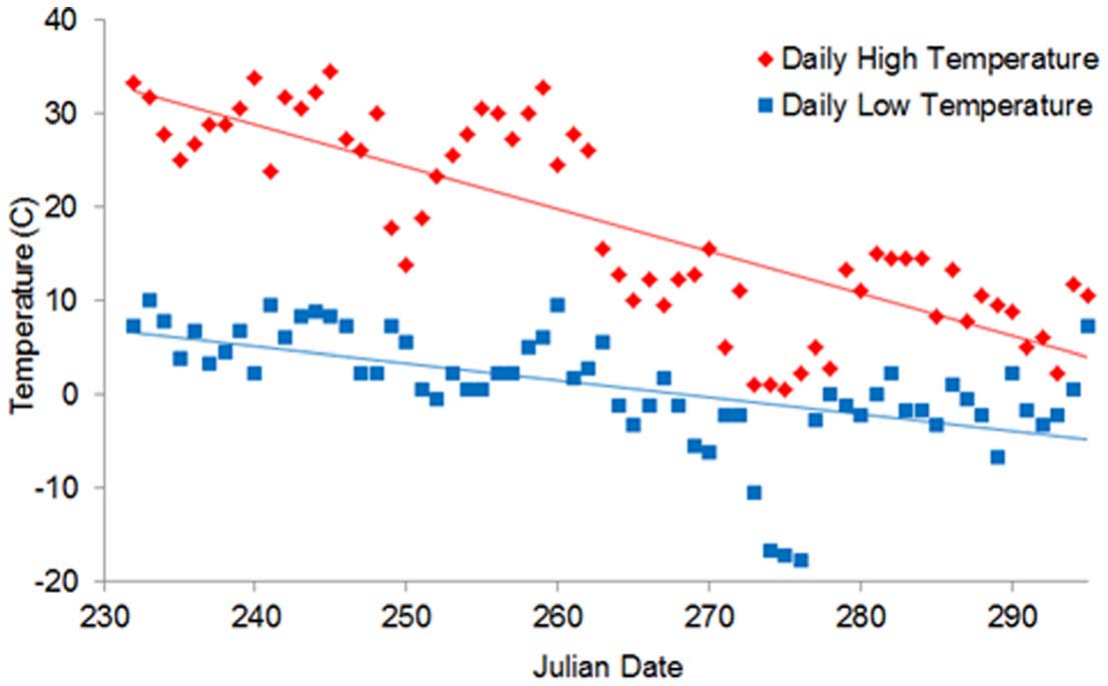
Change in high and low temperature (°C) over the sampling period in West Glacier, Montana, 2001.

The study period was from Julian date 250 (early September), when day time temperatures were 27° to 35°C, until Julian date 300 (end of October), when temperatures were near freezing, ca. 0° to −5°C (Fig. 2). Two trails, approximately 19 km in length each, were surveyed daily to ensure that each new scat found was <24 hours old.

When a fresh scat (<24 hrs) was encountered, the sample was divided in half. One half was mixed thoroughly to equalize the distribution of hormone throughout the sample and stored frozen immediately upon returning from the field (within 4 hrs of collection on average with an 8 hr maximum). This was referred to as the “time 0” subsample. The second half of the sample was left intact to retain its original characteristics until collection, and in its original position in the environment (or moved slightly [<1 m] if at risk of being trampled). The second half, referred to as the “time 1” subsample, was randomly assigned to one of five time exposure groups: 1, 2, 7, 14, or 28 days. Field crews returned to the second half of each sample at the designated exposure time, thoroughly mixed the remaining half sample and stored it frozen, as described above. Although hormones are not evenly distributed in scat [22], we assumed that each sample was sufficiently large that hormone concentrations would be relatively comparable between the sample halves. At the least, hormone concentrations would have varied randomly between the two halves.

We collected the following information about each sample: GPS location, elevation, date, primary and secondary food contents, moisture, odor, odor strength, presence of mold, substrate, and initial exposure conditions. We used date and location information to derive proxies for thermal stress based on average temperatures (Fig. 2) and exposure (i.e., aspect, slope, and elevation). We transformed aspect, which reflects microclimate [23], to a continuous variable ranging from +1 to −1 using the following formula: 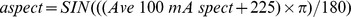. Thus, +1 is the hottest aspect (west-facing) and −1 (NE facing) the coolest. We used a geographic information system to average aspect across every 30 m pixel that intersected a 100 m buffer around each sample’s location. Aspect would be important if bears seek out and compete for these locations to better regulate thermal demands. Our method assumes that fecal sample location is a reasonable proxy of the bear’s average location. Any random error associated with this assumption should decrease rather than increase the significance of the covariates in our model.

All fecal samples were freeze-dried within 30 days post-collection and stored at −80°C until extracted and analyzed for glucocorticoid concentration. In other omnivores, freeze-drying samples and expressing hormone content per g of dry feces has been shown to minimize dietary impacts on fecal hormone excretion rates, independent of nutrition [12].

All samples were extracted and assayed for fGC using the method described by Wasser et al. [9]. Briefly, a 0.2 g subsample of dried, pulverized, well-mixed sample was weighed to the nearest 0.0001 g and transferred to a 15 ml vial. After adding 4 ml of 90% methanol to the sample, the vial was capped and then shaken for 30 min in a pulsing vortexer. Samples were subsequently centrifuged (20 min) and 1 ml of supernatant removed per sample and stored at −20°C until assayed. Extracts were then diluted four-fold in assay buffer and quantified for fGC with a double antibody ^125^I radioimmunoassay kit (MP Biomedicals [previously ICN], Solon, OH, catalog #07-120103). We used the manufacturer’s protocol except with half-volumes throughout. Low and high controls were included in every assay. Non-specific binding tubes and blanks were assayed in quadruplicate, and standards, controls, and samples in duplicate; any sample with a CV>10% between duplicates was re-assayed to confirm results. This assay has previously been validated for black and grizzly bear fGC [9]. For other assay details, see Wasser et al. [9] and Hunt and Wasser [11].

### Statistical Analyses

Fecal glucocorticoid measures were normalized by log transformation (log10(X+1)) in all analyses. We used generalized linear models (GLM) to first predict fGC concentrations in fresh (time 0) samples based on proxies of nutrition (berry and meat>vegetation [24]), ambient temperature (Julian date; Fig. 1), and thermal exposure (e.g., aspect, slope, elevation, and heat load index). Degradation effects were assumed to be negligible in the time 0 samples (≤24 hrs old) because previous work [11] showed GCs in bear scat to be highly stable. We found support for this assumption by showing that precipitation (from time of defecation to sample collection) had no effect on fGC concentrations of the time 0 samples after discovering precipitation to be the principle cause of degradation in the time 1 samples (see results). The covariates from the time 0 GLM were next used to predict fGC concentrations in the exposed sample halves to determine whether the same environmental covariates still reliably predict fGC concentrations, with and without including effects of exposure time and precipitation. All variables included in our final models were derived from two separate GLMs, support for which was based on AIC_c_ values [25].

All significant variables in these two GLMs were then included in a repeated measures multivariate analysis of variance (MANOVA) to identify the environmental variables that affected both between-sample variation in fGC (independent of exposure time), and the within-sample degradation effects of exposure time. This enabled us to determine whether degradation impacts were severe enough in the time 1 subsamples (i.e., the sample halves that were left in the field for 1–28 days) to mask differences in fGC levels due to environmental stressors detected in time 0 (fresh) subsamples.

Effect size was also examined in the MANOVA analyses to compare the whole models as well as all variables included in each model, using Cohen’s 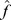 statistic, where:
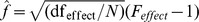



By convention, effect sizes of 0.02, 0.15, and 0.35 are termed small, medium, and large, respectively.

## Results

### Analysis 1: Factors Influencing fGC Levels at Time 0

**Figure 3:**
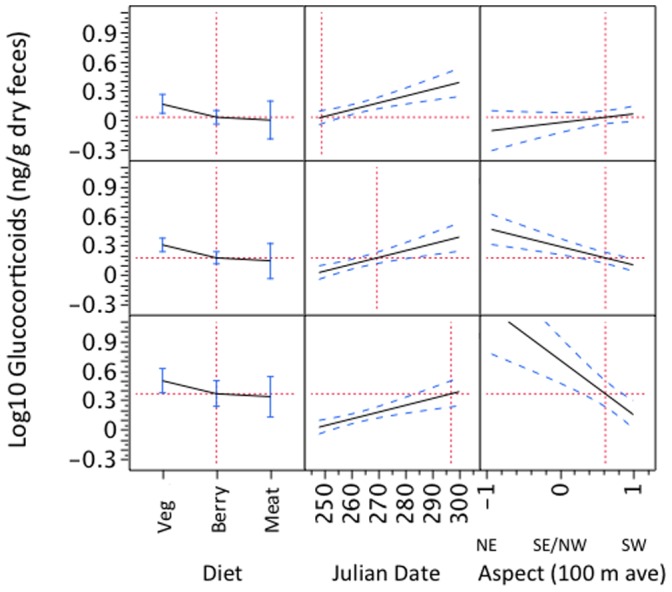
Effect of diet, Julian date, and aspect on glucocorticoid concentrations in time 0 bear scats collected in Glacier National Park, Montana. Each row reflects a different Julian date, 20 days apart, as indicated by the dashed vertical line in the center column of each row. Aspect is transformed, whereby −1 =  coolest and +1 =  warmest aspect (see text).

The main effects explaining variation in fGC concentrations at time 0 (i.e., prior to degradation effects), based on AIC_c_ model support, were food contents (vegetation vs berries or meat), Julian date, aspect, as well as an interaction between Julian date and aspect (Table 1). Time 0 fGCs increased with Julian date, being lowest at earlier dates (warmer temperatures) and highest at later dates (colder temperatures; Fig. 2). The interaction with aspect, however, indicated that fGCs were lowest in cooler aspects at earlier dates (higher temperatures), and in warmer aspects at later dates (colder tempteratures). Essentially, fGCs were lower in scats collected in cool locations (aspects) during hot weather and in warm locations during cold weather (Fig. 3).

**Table 1:** Model estimates and standard errors for impacts of diet, Julian date, aspect, and precipitation on glucocorticoid concentrations [log10(x+1)] in fresh bear fecal samples collected in Glacier National Park, Montana.

Variable	Time 0	Time 0	Time 1	Time 1
**Sample Size**	41	41	41	41
**AICc**	−36.78	−30.91	−14.04	−17.44
**Diet: Veg vs Meat** **Berry vs Meat**	0.10±0.041–0.03±0.037	0.1±0.041–0.032±0.037	0.123±0.054–0.098±0.049	0.14±0.052–0.091±0.044
**Julian Date (Temperature)**	0.007±0.0018	0.007±0.0018	0.0045±0.002	0.006±0.0024
**Aspect**	−0.13±0.039	−0.107±0.068	−0.078±0.052	−0.075±0.074
JulianDate*Aspect	−0.0132±0.0037	−0.0135±0.0037	−0.0126±0.0048	−0.0143±0.0044
**Precipitation (cm)**		−0.084±0.12		−0.03±0.011
Precip*Aspect		0.188±0.48		0.067±0.032

Time 0 fGCs also appeared to reflect nutritional quality of the diet; fGCs were highest for scats containing primarily vegetation (grass, leaves, stems, roots, tubers, and corms, presumably indicating relatively poor nutrition and/or high stress) and lowest for scats containing berries and meat (implying relatively good nutrition and/or low stress; Table 1).

As expected, precipitation had no effect on fGC concentrations in the time 0 samples, supporting our assumption that degradation effects were undetectable in the time 0 samples (Table 1).

### Analysis 2: Factors Influencing fGC Levels at Time 1

We examined fGC stability over time by assessing whether the same variables predicting fGC concentrations in the time 0 samples remained significant in the time 1 samples, with or without the addition of all degradation measures (e.g., exposure time, precipitation, and their interactions with aspect). All predictors of fGC in the time 0 samples remained significant predictors in the time 1 samples. Adding precipitation and its interaction with aspect, however, significantly improved the model predicting fGC concentrations in the time 1 samples (Table 1). Increased cumulative precipitation between time 0 and time 1 decreased fGC concentrations of the time 1 samples and the cooler the aspect, the more this effect was exacerbated.

### Analysis 3: Covariates Influencing GC Degradation

The repeated measures MANOVA was consistent with the above findings (Table 2). Diet, Julian date, and the interaction between Julian date and aspect remained significant predictors of between-sample variation in fGC. Cumulative precipitation, which significantly affected fGC concentrations in the time 1 but not time 0 samples, was also a significant predictor of between-sample variation in fGC in the MANOVA. However, only diet and the interaction between cumulative precipitation and aspect were significant predictors of within-sample variation over time. Cumulative precipitation in samples exposed in cooler aspects showed increased degradation over time (Table 2).

**Table 2:** Results from a repeated measures MANOVA, showing between- and within- sample effects of diet, Julian date, aspect (100 m ave), precipitation and their interactions with exposure time, on fecal glucocorticoid concentrations [log10(x+1)].

	DF	Exact F, N = 41	P	Effect Size
All-Between	7	6.028	0.0001	0.927
Intercept	1	8.004	0.008	0.413
Diet	2	5.794	0.007	0.484
Julian Date	1	10.257	0.003	0.475
Aspect	1	0.0001	0.99	0.000
JD*ASP	1	11.629	0.002	0.509
Cumul Precipitation (cm)	1	6.396	0.016	0.363
All-Within Interactions	7	1.962	0.091	0.405
Exposure Time (ET)	1	2.1	0.157	0.164
ET *Diet	2	3.387	0.046	0.341
ET*Julian Date (JD)	1	2.037	0.163	0.159
ET*Aspect (ASP)	1	6.91	0.013	0.380
ET*JD*ASP	1	0.0274	0.87	0.000
ET*CumulPrecip	1	1.131	0.3	0.057
ET*Aspect*CumulPrecip	1	3.95	0.055	0.268

Time in this analysis reflects the period between collections of each time 0 and time 1 sample. Effect sizes are also shown based on Cohen’s 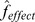, where 0.02, 0.15, and 0.35 represent small, medium, and large effects, respectively.

## Discussion

The biological effects of temperature (Julian date) and thermal exposure (aspect) on bear fecal hormones at time 0 suggested that fGC may be a good indicator of thermoregulatory demands in ursids. In particular, fGC increased linearly with Julian date, indicating increased fGC in the fall as ambient temperatures declined. Similar effects of elevated cortisol levels related to thermoregulation have been described for other mammals [13], [14]. The interaction between Julian date and aspect also suggests that fGC during the hottest temperatures were lowest in bears that presumably spent more time in the coolest aspect. Similarly, fGC in the coldest temperatures were lowest in bears that spent more time in the hottest aspect. The colder the temperature, the greater the effect that aspect had on fGC levels. This reversal in effect of aspect on fGC would not be expected if Julian date were simply reflecting fGC degradation occurring up until the time 0 sample was first located (i.e., ≤24 hours post defecation). If degradation resulted from hotter ambient temperature, fGCs should be lower, not higher, in samples collected in the hotter aspect during the hottest time of year. If degradation resulted from cooler temperatures and/or greater precipitation, fGCs should be lower, not higher, at later Julian dates and particularly in cooler aspects at that time. Degradation effects from precipitation were also ruled out in these time 0 samples. Overall, our data suggest that bear fGC concentrations may be affected by thermoregulatory demands. These patterns suggest that fGC measures should be investigated as a potential technique for landscape-wide assessments of thermoregulatory load in free-ranging animals, for example, in studies of climate change and related effects.

We also found a significant effect of diet on fGC concentrations at time 0, indicating that fGC concentrations also corresponded to diet quality in ursids, as has been found in other vertebrates [2], [5], [10], [16], [17]. We found that fGC concentrations were highest for the least nutritious diet (coarse vegetation) and lowest for the most nutritious diets (berries and meat). An alternative possibility is that dietary fiber content might affect steroid excretion rates directly via changes in gut transit time or fecal mass. We controlled for excretion rate effects by freeze-drying samples prior to extraction, as this has proved effective in other omnivores [12]. Moreover, berries and vegetation both contain high amounts of dietary fiber compared to meat [25], but only vegetation had elevated fGC. Gut transit time is also faster in grizzly bears fed vegetation diets compared to meat diets [25], which would tend to decrease fGC in vegetation-based scats, yet we observed an increase. We therefore conclude that the patterns we observed reflect nutritional impacts on circulating GC as opposed to effects of gut transit time or fiber content on fGC excretion rates per se. Vegetation diets are known to be a less preferred diet for grizzly bears [24], [26], and vegetation diets have substantially lower protein content and digestibility [25]. Vegetation-based diets appear to represent a nutritional stressor for grizzly bears that is accurately reflected in fGC content of fecal samples.

It is possible that the part of the between-sample variance in fGC explained by Julian date was also due to seasonal changes in diet, as nutritionally important berries were most abundant earlier in the sampling season when fGC was lowest. Such nutritional effects would not, however, explain the interaction between Julian date and aspect on fGC; those can only be reconciled by the joint effects of temperature and exposure. Further, scats had a mixture of high and low quality food items throughout the sampling season. For example, highly nutritious berries were the primary contents in some scats as late as mid-October.

Some fGC degradation did occur over the 28 day exposure period, largely due to cumulative precipitation, particularly in cooler aspects where evaporation would be reduced. Overall, fGC declined in the time 1 samples as a function of aspect interacting with precipitation. Yet in time 0 samples, this relationship was reversed; fGC increased in time 0 samples when aspect interacted with Julian date (cold temperatures), despite greater precipitation at later Julian dates. These differing relationships in time 0 vs. time 1 samples strongly support our conclusions that (a) fGC reflects thermoregulatory demands and (b) that some fGC degradation does occur in samples exposed to cumulative precipitation for periods of weeks prior to collection. These latter effects, however, were still insufficient to mask the effects of the biological covariates predicting fGC concentrations in the time 1 samples.

Finally, autocorrelation of fGC measurements is unlikely to contribute to study results because multiple samples were collected in close proximity to each other on only one occasion (Fig. 1). Future field studies could genotype scat as an added safeguard against autocorrelation, as done by Wasser et al. [1].

### Conclusions

Scat can provide a wide variety of physiological and genetic information and is the most accessible biological product from wildlife in nature. Collectively, this provides the opportunity to partition impacts from a number of multiple environmental pressures, given the right study design [5], [10]. Our results suggest that fGC analyses can provide insight about thermoregulation and nutrition in grizzly bears and black bears, and may serve as an index of physiological response to climate change. We further showed that some sample degradation impacts do occur in the wild. These effects were, however, insufficient to mask detection of the biological environmental impacts on ursids in samples exposed for up to one month. Regardless, controlling for cumulative precipitation over the sample exposure period can be used as an added precaution against such degradation impacts masking biological effects. Similar studies examining other hormones, environments, diets, and species will be important to evaluate how far our findings can be generalized.
